# The inhibition of invasion of human melanoma cells through N-cadherin knock-down

**DOI:** 10.1007/s12032-018-1104-9

**Published:** 2018-02-28

**Authors:** Dorota Ciołczyk-Wierzbicka, Piotr Laidler

**Affiliations:** Chair of Medical Biochemistry, Jagiellonian University Medical College, ul. Kopernika 7, 31-034 Kraków, Poland

**Keywords:** Melanoma, Cell invasion, N-cadherin, Protein kinase inhibitors, siRNA

## Abstract

N-cadherin seems to promote cell migration and invasion in many types of cancers. The object of this study is recognition of the possible role of N-cadherin and selected downstream protein kinases: PI3K, ERK1/2, and mTOR in cell invasion in malignant melanoma. Melanoma cells were transfected with the small interfering RNA (siRNA) that targets human N–cadherin gene (*CDH2*). Inhibitors LY294002 (PI3K), U0126 (ERK1/2), and everolimus (mTOR) were used to inhibit selected kinases of signalling pathways. In vitro cell invasion was studied using Matrigel and an analysis of matrix metalloproteinases MMP-2 and MMP-9 activity by gelatinase zymogram assay. Treatment of melanoma cell with either siRNA against N-cadherin or protein kinase inhibitors led to significantly decreased MMPs expression and activity, as well as diminished invasion. Both the current and the former results suggest that activation of PI3/AKT, mTOR, and ERK kinase, following N-cadherin expression, contributes not only to increased proliferation but also invasive potential of melanoma cells. The results also indicate that N-cadherin, as well as the studied kinases, should be considered as a potential target in melanoma therapy.

## Introduction

It is becoming ever clearer that integration of signals triggered by cell–cell interactions, cell–matrix signalling, and growth factor signalling plays a crucial role in cellular behaviour. Cadherins have been implicated in a number of signalling pathways that stimulate cellular behaviour. They are also of key importance for embryo compaction [1], cell intercalation [2, 3], and cell sorting [4]. Furthermore, cadherins with associated protein-mediated cell–cell adhesion promote a unique cytoskeletal structure that supplies adhesive strength [5–9]. Interest in cadherins has grown since the late 1980s, when they were found to be linked with cancer progression and other diseases.

Human melanocytes express both E-cadherin and P-cadherin. E-cadherin is responsible for adhesion of melanocytes to keratinocytes. Melanocytes embedded in the basal layer of epidermis interact with keratinocytes through E-cadherin, which in effect regulates their growth [10]. Pathologic changes leading to growth of malignant melanomas disturb the delicate homeostatic balance between melanocytes and keratinocytes and can lead to altered cell–cell adhesion and cell–cell connections. In majority of epithelial cancers, loss of E-cadherin (*CDH1*), P-cadherin (*CDH3*) or both during tumour progression results in an increased expression of the mesenchymal cadherin, i.e. N-cadherin, with a significant change in the adhesive properties of cancer cells, as they lose their affinity for epithelial neighbours in favour of stromal cells [5, 11–15].

One of the first and most important steps in the metastatic cascade is the loss of cell–cell and cell–matrix interactions. N-cadherin, a crucial factor of homotypic and heterotypic cell–cell interactions, might play a central role in the metastasis.

It was shown that in various cancer cells expression of N-cadherin is associated with cell migration as it induces cells’ motility in many types of cancers such as breast, prostate, and gastric cancer. Since overexpression of N-cadherin increases migration and invasion of cells without reducing the level of E-cadherin, these results suggest that it is the presence of N-cadherin rather than the absence of E-cadherin that is instrumental for increased cell invasiveness [16]. Therefore, strict regulation of N-cadherin expression is essential in normal epithelial cell function [16].

Degradation of basement membranes and stromal extracellular matrix (ECM) is crucial for invasion and metastasis of malignant cells. Extracellular matrix degradation is initiated by proteinases secreted by different cell types participating in tumour cell invasion, and increased expression of every known class of proteinases has been linked to malignancy and invasion of tumour cells [17]. Gelatinase A (MMP-2, 72kDa gelatinase) is expressed in a variety of normal and transformed cells, including fibroblasts, keratinocytes, endothelial cells, and chondrocytes. Gelatinase B (MMP-9, 92kDa gelatinase) is produced by keratinocytes, monocytes, macrophages, and many malignant cells. High levels of MMP-9 were detected in breast, colorectal, and gastric cancers. The possibility of using MMP-9 as a marker of skin melanoma vertical growth phase (VGP) has been suggested [18].

In addition to induction of degradation of extracellular matrix components, MMP-9 is involved in regulation of the basic biological processes of: apoptosis, proliferation, and differentiation [19, 20].

The role of N-cadherin in cell differentiation, cancer transformation, and invasion has also been documented [11, 21] with some of these functions, most probably depending on the activation of intracellular signal transduction cascades [22]. However, neither the mechanism by which N-cadherin regulates entry into the cell cycle nor its migratory characteristics have yet been fully elucidated.

Therefore, following our earlier report on the role of N-cadherin in melanoma proliferation [23], we sought to look at the possible initiative role of this adhesion molecule on cancer cell migration and invasive potential.

## Materials and methods

### Cell culture

Human melanoma cell lines: WM793 (VGP—vertical growth phase); Lu1205 (metastatic; biopsy taken from the lung; selection in mice; a culture from the primary site (sternum area) from the same donor as WM793. WM115 (VGP—vertical growth phase) and WM266-4 (metastatic, skin) were cultured in RPMI-1640 medium supplemented with 10% foetal bovine serum and antibiotics: penicillin, streptomycin. Cells were incubated at 37^◦^C in a humidified atmosphere of 5% CO_2_ in air. Cells were treated with inhibitors of: 1/PI3K—LY294002 (Cell Signalling TM) at 20 μM concentration, 2/ERK1/2—U0126 (Cell Signalling TM) at 10 μM concentration, and 3/mTOR—everolimus (Selleck) at 5 nM concentration. The incubation time of melanoma cells with inhibitors was 24 and 48 h. Cells were obtained from the ESTDAB Melanoma Cell Bank (Tubingen, Germany).

### siRNA transfection of melanoma cells

Melanoma cells were grown until 60% confluence was reached, and then transfected using Oligofectamine reagent (Invitrogen), in keeping with the manufacturer’s protocol, previously described [23] 21 bp double-stranded siRNA molecules specifically targeting for the N-cadherin: siRNA *CDH2* (target sequence 5′-AAAGTGGCAAGTGGCAGTAAA-3′—nucleotides 798–818; NM001792)—generated in vitro transcription (SilencerTM siRNA Construction Kit (Ambion)) and commercially available (Ambion ID#S27773) or a control non-silencing sequence (Ambion ID#4390843) [23]. The effects that RNA interference had on expression of CDH2 mRNA and protein were determined by reverse transcription-PCR (RT-PCR) and Western immunoblotting, respectively, described previously [23].

WM793, WM115, and WM266-4 cells were transfected with 50 nM, and Lu1205 cells with 100 nM siRNA. Medium was replaced 24 h later with a fresh one, and cells were grown for an additional 24 to 48 h period (48 or 72 h post-tansfection) prior to further analysis [23].

### Cell migration and invasion assay

Cell invasion assays were performed using conventional Boyden transwell methods in keeping with the manufacturer’s protocol (BD BioCoat™ FluoroBlok Invasion System No. 354166). Melanoma cell migration was studied with the use of FluoroBlok Inserts System No. 351157. Quantitation of invasion was achieved by post-invasion cell labelling with Calcein AM (Fluka), and measuring the fluorescence of invading cell samples at excitation/emission wavelengths of 485/530 nm sensitivity 100 on a plate reader (BIO-TEK).

### Zymography

Melanoma cells from primary (WM793, WM115) and metastatic sites (Lu1205, WM266-4) were grown as monoculture for various periods of time (24, 48 h). Serum-free, conditioned media were collected, and activity of the metalloproteinases: MMP-2 and MMP-9 was analysed using gelatin zymography.

Gels were prepared in the presence of 0.1% gelatin (Sigma) in non-reducing conditions. Proteins were loaded per well and separated with the use 4.5% stacking and 10% separation gel in a 4 h run. Following electrophoresis, the gel was washed two times for 30 min in 2.5% TritonX-100.

The gel was intubated at 37 °C for 48 h in buffer (50 mM Tris pH 7.5; 10 mM CaCl_2_; 0.15 mM NaCl).

Then the gel was stained in a solution containing 1% Coomasie blue R250 in 50% methanol and 10% acetic acid for 1 h. Gelatinolytic activity was observed as clear areas in the gel.

### Densitometry analysis

Densitometry analyses of gelatinolytic activities of metalloproteinases MMP-2 and MMP-9 were performed on raw volume (sum of intensities of bound—volume calculated from the area of the peak) using SynGene Gene Tools version 4.03.0 (Synoptics Ltd Beacon House, Nuffield Road Cambridge, CB4 1TF, UK).

### Cytotoxicity assay

Cytotoxicity of N-cadherin siRNA (100 nM), PI3K inhibitor—LY294002 (20 μM), ERK1/2 inhibitor—U0126 (10 μM), and everolimus (5 nM) mTOR inhibitor assay was determined using Cytotoxicity Detection Kit LDH, Roche, Germany. In all examined melanoma cell lines, both N-cadherin siRNA and inhibitors: everolimus, LY294002, U0126 showed no cytotoxicity effect when tested in a culture medium at the time of 72 h. LDH activity in the culture medium in no case exceeded 3%.

### Western blot analysis

Preparation of samples for electrophoresis and western blot analysis was made as described previously [23]. The following antibodies: MMP-2 (AV20016, SIGMA), MMP-9 (IM09L, Merck Millipore), N-cadherin (610920, BD Transduction Laboratories) and β-actin (A2228, SIGMA) were used.

### Statistics

Cell invasion data were calculated from mean values of repeated experiments. Statistical analyses were performed using one-way ANOVA with post hoc Tukey test (Statistica 12.0 StatSoft); asterisk (*) indicates a significant difference *p* < 0.005, and double asterisk (**) indicates a significant difference *p* < 0.0005.

## Results

### N-cadherin regulates cell invasion

The study on the role of N-cadherin in migration and invasion of melanoma cells was performed using conventional Boyden transwell methods. All tested melanoma cell lines manifested the ability for cellular migration as a capacity to chemotaxis. Cells’ invasion after 48-h knock-down of N-cadherin compared with control (non-specific siRNA) or non-treated cells was measured.

**Fig. 1:**
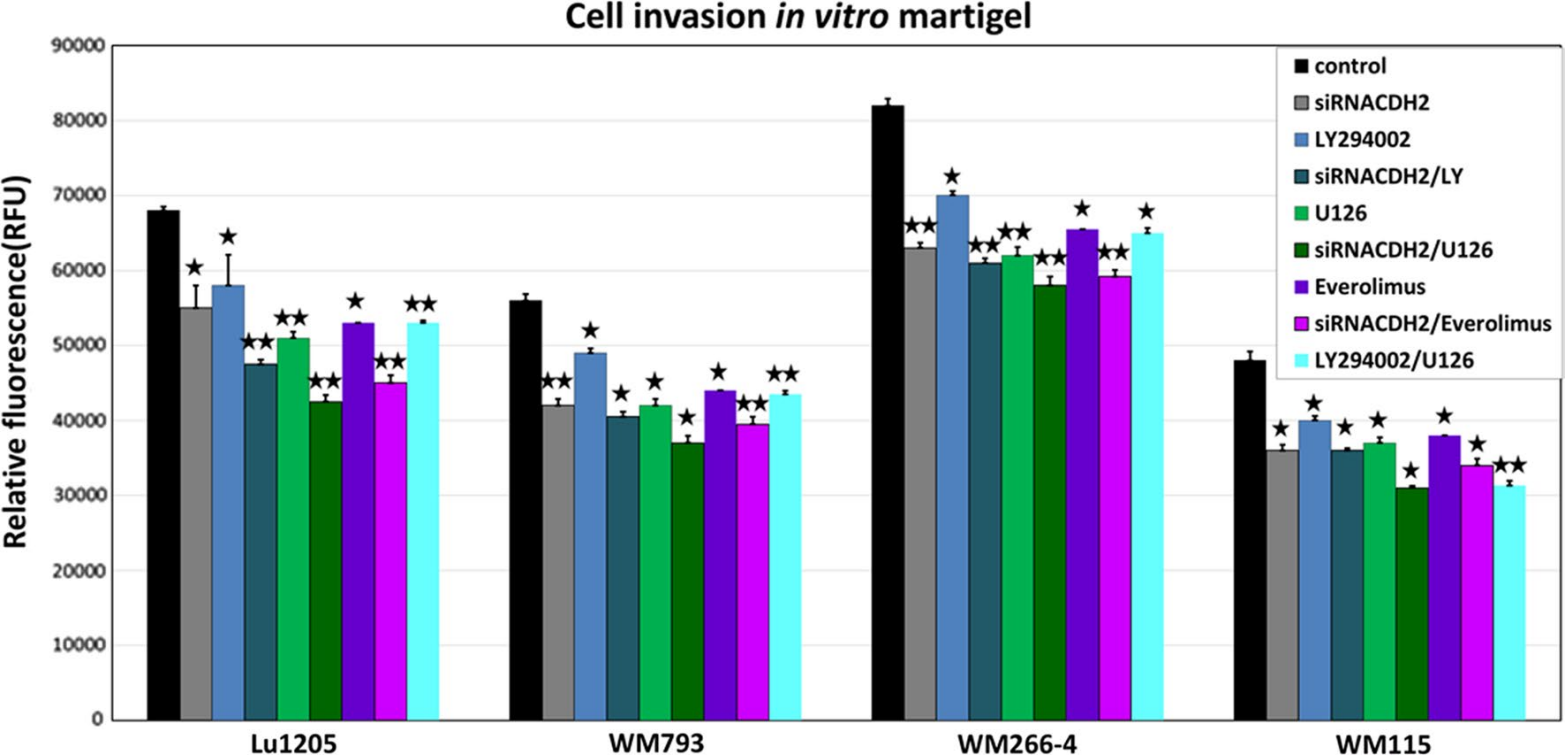
The effect of N-cadherin silencing by siRNA and protein kinase inhibitors on melanoma cell invasion “in vitro”. Cell invasion assay through Matrigel-coated Boyden chamber. Histogram shows the quantification of cell invasion. Values are expressed as mean ± standard deviation in 4 wells in two independent experiments. All results are presented as experimental mean values which were compared using one-way ANOVA with the Tukey’s post hoc test (Statistica ver. 12, StatSoft,); asterisk (*) indicates a significant difference: *p < 0.005, **p < 0.0005

N-cadherin siRNA-transfected cells showed reduction in invasion by 20–25% when compared with control cells (Fig. 1). The effect was observed for primary WM793 and WM115, as well as metastatic Lu1205 and WM266-4 cell lines. The most inhibition was, noticed in case of WM793 (VGP) and WM266-4, invasion of which was reduced by: 25% (*p* < 0.0005) and 23% (*p* < 0.0005), respectively (Fig. 1).

Separate use of inhibitors: U0126 (ERK1/2) or everolimus (mTOR) reduced melanoma cell invasion by approximately 21–25% (*p* < 0.005), whereas treatment with LY294002 (PI3K) reduced it by only about 15% (*p* < 0.005). Treatment of melanoma cells with combination of U0126 (ERK1/2) and LY294002 (PI3K) inhibitors decreased it by about 25% (*p* < 0.005). Applications of a combination of siRNA for N-cadherin and U126 (ERK1/2) inhibitor resulted in a reduction of invasiveness of cells by about 38% (*p* < 0.0005) in Lu1205 cell line, and similar response was observed in the case of a combination of siRNA for N-cadherin with everolimus (mTOR) inhibitor (Fig. 1).

### N-cadherin regulates gelatinolytic activities of the metalloproteinases MMP-2 and MMP-9

**Fig. 2:**
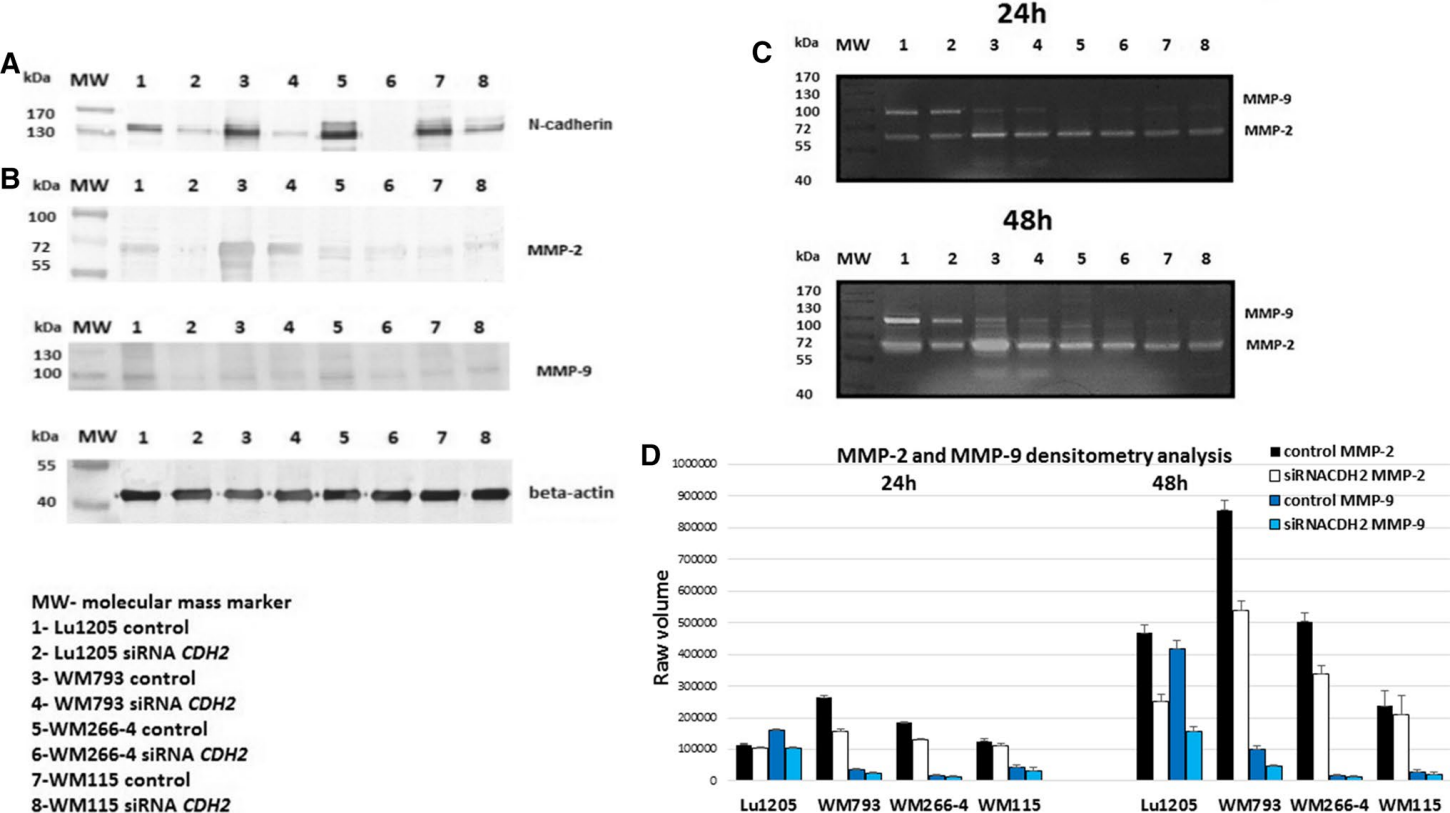
N-cadherin regulates and MMPs activity. **a** The effect of N-cadherin silencing in melanoma cells. **b** Identification of the metalloproteinases 2 and 9 in melanoma cell lines by western blot analysis. **c** The effect of N-cadherin silencing on gelatinolytic activities of MMP-2 and MMP-9 in melanoma cells. **d** Densitometric analysis of MMP-2 and MMP-9 activity. Raw volume (sum of intensities of bound—volume calculated from the area of the peak)

siRNA for the N-cadherin (siRNACDH2) effectively reduced the expression of N-cadherin (Fig. 2a). In a previous work, [23] described in detail the effect of silencing N-cadherin on melanoma cells.

The invasive ability of cancer cells can be regulated through expression of zinc-dependent endopeptidases, MMPs. Metalloproteinases 2 and 9 were identified in melanoma cell lines through western blot analysis (Fig. 2b). High protein levels of metalloproteinase MMP-2 were found in all examined melanoma cell lines. In the case of metalloproteinase MMP-9, a much lower protein level was observed except in Lu1205 cell line (Fig. 2b).

Therefore, activities of MMP-2 and MMP-9 were studied in both the primary (WM793, WM115) and metastatic (Lu1205, WM266-4) cells lines. MMP-9 activity was always much lower than that of MMP-2, except for the Lu1205 cells. Both monomeric and dimeric forms of MMP-9 were observed in all tested cell lines except Lu1205 (Fig. 2c).

Zymography used to measure the activity of metalloproteinases in melanoma cells revealed a significant difference between MMP-2 and MMP-9 activities in N-cadherin knocked-down cells in all tested cell lines in comparison with untreated ones. The greatest decreases in MMP-2 activity were observed after 48-h treatment. These reached about 47% in the Lu1205 cells, and about 40% in WM793 line. The smallest decreases, at the level of 12%, were observed in WM115. In the case of MMP-9, decreases of activity reached 62 and 52% of their initial values in Lu1205 and WM793 cells, respectively. Summary of the results of densitometry analysis of the activity of MMP-2 and MMP-9 after N-cadherin knock-down is presented in (Fig. 2d).

We sought to check if the decreases in MMPs activities caused by N-cadherin silencing may be strengthened by additional inhibition treatment with selected signal kinases inhibitors.

**Fig. 3:**
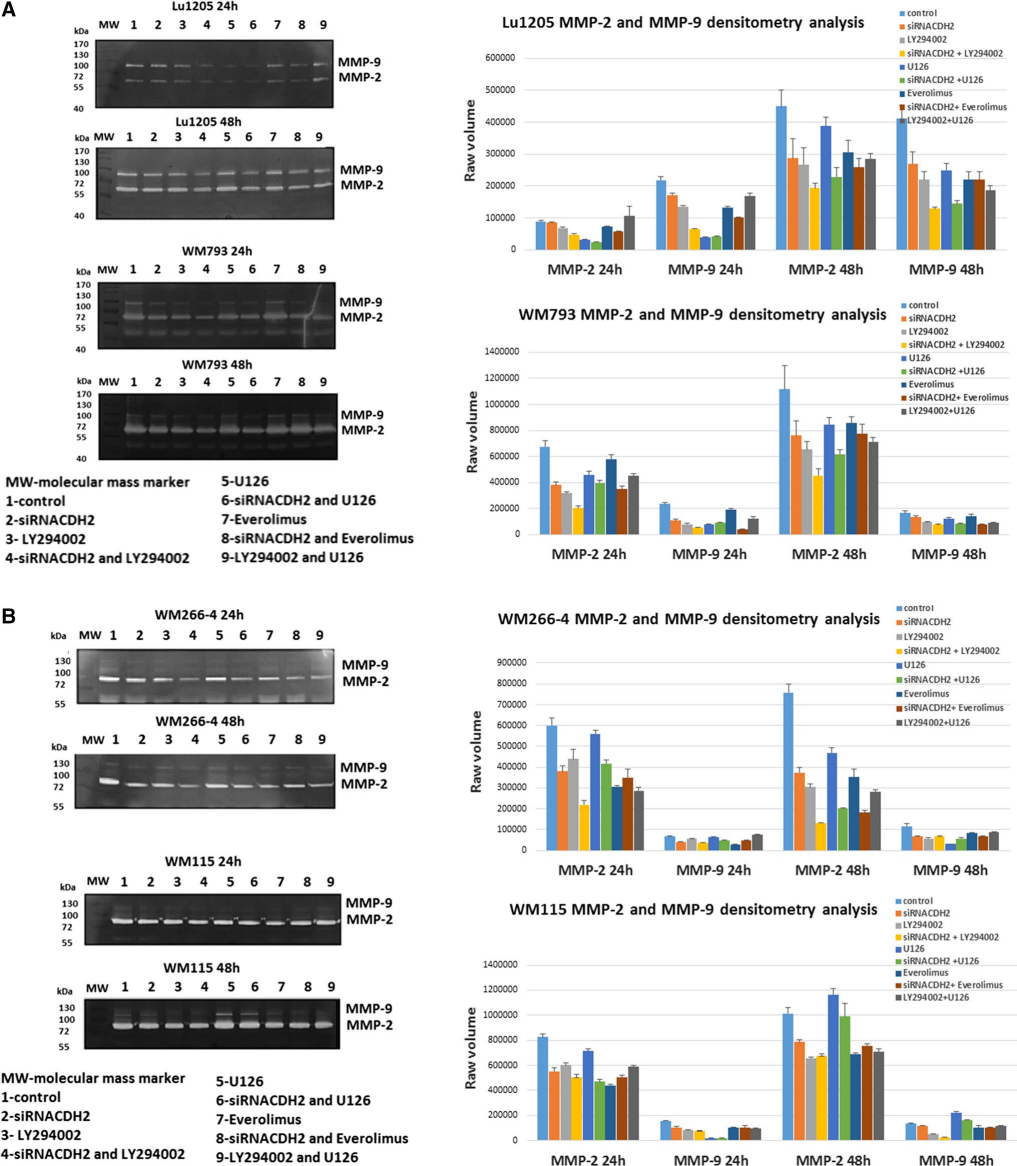
The effect of N-cadherin silencing and protein kinase inhibitors on gelatinolytic activities of MMP-2 and MMP-9 in melanoma cells: **a** Lu1205 and WM793. **b** WM266-4 and WM115. Densitometric analysis of MMP-2 and MMP-9 activity. Raw volume (sum of intensities of bound—volume calculated from the area of the peak)

The largest decreases in the activities of metalloproteinases, particularly in MMP-2, were observed in the case of 48-h treatment of cells with combination of siRNA for N-cadherin and PI3K inhibitor—LY294002 (Fig. 3a, b). With shorter (24 h) incubation time, best results were achieved with application of combination of siRNA for N-cadherin and inhibitor U0126 (ERK1/2). High efficiency of MMPs inhibition was also reached with the combination of siRNA for N-cadherin and inhibitor everolimus (mTOR), regardless of incubation time. Use of a single inhibitor gave similar results to N-cadherin gene silencing, but much less promising than in the case of combinations of inhibitors and siRNA for N-cadherin (Fig. 3a, b).

The activity of MMP-9 was much lower than that of MMP-2, with the exception of Lu1205. As in the case of MMP-2, the greatest decreases in MMP-9 activity were observed when using a combination of siRNA for N-cadherin and PI3K inhibitor—LY294002. Similar results were observed for both metalloproteinases: MMP-2 and MMP-9 after application of mTOR inhibitors—everolimus. Melanoma cells react differently to MEK kinase inhibitor. Small increase in the activity of metalloproteinases in the two cell lines: WM115 (VGP) and WM 266-4 (from the same source) was observed.

Figure 3a, b illustrates the results of densitometry comparison of MMP-2 and MMP-9 activity after knock-down of N-cadherin and treating with U0126 (ERK1/2), LY294002 (PI3K) and everolimus (mTOR) inhibitors.

## Discussion

It has been previously demonstrated that N-cadherin had significant effect on cell signalling, cell cycle regulation, and, in consequence, proliferation of melanoma cells, as the use of siRNA against N-cadherin led to significant reduction in number of such treated cancer cells [23].

The current study confirmed that melanoma cells transfected with N-cadherin specific siRNA successfully and transiently decreased its expression at mRNA and protein levels. This resulted in inhibition of metalloproteinase 2 and 9 activities, as well as reduced cell invasion.

In various cancer cells, high expression of N-cadherin is associated with activation of cell motility. Expression of N-cadherin induces cell migration of cancer such as breast, prostate, gastric, and melanoma [21, 24–27]. Furthermore, the E-cadherin to N-cadherin switch is often found in aggressive cancers [28]. The up-regulation of N-cadherin in aggressive carcinomas suggests that the level of N-cadherin expression is a critical step for cancer cell invasion.

### Melanoma cell invasion

The results of the study on the effects of N-cadherin knock-down on cell invasion confirm the role of this adhesion molecule in the process of invasion of melanoma cells. In all tested melanoma cell lines, a statistically significant decrease in the number of invading cells in the range between 20 and 25% was observed. Furthermore, impact of protein kinase inhibitors: LY294002, U126, everolimus, and combination of siRNA for N-cadherin and protein kinase inhibitors on the process of melanoma cell invasion reported here suggests involvement of all three kinases in N-cadherin-initiated signalling. Simultaneous administration of any of protein kinase inhibitors and siRNA for N-cadherin resulted in a decrease of approximately 30%, whereas with the use of a combination of siRNA for N-cadherin and ERK MAPK, pathway inhibitors-U126 diminished cells invasion by up to 38%.

### MMPs in tumour progression

MMPs play an important role in many biological and pathological processes. Uncontrolled, activity of metalloproteinases may well lead to development of many diseases such as arthritis, atherosclerosis, aneurysms, nephritis, tissue ulcers or fibrosis, and cancer [29]. The MMPs have been present in the discussion on cancer for more than 40 years, being overexpressed in a large range of malignant tumours in response to oncogenic transformation, and activation of cytokines, several growth and angiogenic factors [30].

Initial observations on the role of MMPs in the cancer biology have suggested that the ability of tumour cells to metastasize correlates with increased levels of metalloproteinase activity. Elevated levels of gelatinases, MMP-2 and MMP-9, are often observed in malignant cancers. Among human melanoma cells, MMP-2 and MMP-9 have attracted attention in the recent years, especially with regard to cutaneous, eye, and oral melanomas [30]. Expression of MMP-2 has frequently been associated with malignant progression and poor prognosis [31]. Particularly, high levels of MMP-2 were observed in WM793 melanoma cell line from the primary VGP.

Results of studies using tissue microarray, immunohistochemistry of melanoma biopsies of primary and metastatic lesions, as well as nevi, confirmed that MMP-2 is predictive of primary and metastatic stages [30]. High MMP-2 expression in primary lesion contributes to invasiveness of primary tumour cells, leading to metastases and poor survival outcomes [32].

Similarly to other publications [33], this study showed a high level of the MMP-2 activity in all tested melanoma cell lines.

The second gelatinase MMP-9 proved to be much less active than MMP-2, except for the Lu1205 from metastatic cell line, where activity of MMP-9 was at a high level. Here, monomeric and dimeric forms of MMP-9 were detected in all tested lines except for Lu1205. MMP-9 also exists as a monomeric and homodimeric molecule, in both its latent and active forms. Both monomeric and dimeric forms of MMP-9 have been identified in biological fluids and tissues, in a variety of normal and neoplastic cells [34]. Dimerization or multimerization is mediated by the carboxyterminal domains of MMP-9 and occurs intracellularly [34]. The functional biological role of the MMP-9 dimer has not yet been elucidated; however, dimerization significantly decreases the activation rate of pro-MMP-9 by stromelysin (MMP-3) [34].

The herein reported significant decrease in MMP-2 and MMP-9 protein levels and activities that was observed after knock-down of N-cadherin remains in agreement with the view on the importance of this adhesion molecule for activation of matrix metalloproteinases, and in effect stimulation of invasion and metastasis. The largest decreases of MMP-2 activity were observed in the Lu1205 and line WM793 (by about 47 and 40%, respectively). Concurrently, MMP-9 activity in the cell lines dropped by about 62 and 52%, respectively.

Decreases of MMP-2 and MMP-9 activities were also observed upon using a combination of siRNA for N-cadherin and protein kinases inhibitors: LY294002 (PI3K), U126 (ERK1/2), and everolimus (mTOR). The largest decrease in activity of metalloproteinase, MMP-2 in particular, was observed upon using a combination of siRNA for N-cadherin and PI3K inhibitor LY294002 after 48-h treatment, while at shorter incubation time (24 h) application of a combination of siRNA for N-cadherin and inhibitor U0126 produced best results. Using the combination of siRNA for N-cadherin and inhibitor of mTOR—everolimus gave similar results regardless of the incubation time.

Hazan et al. [21] suggested that N-cadherin functionally interacts with the FGF receptor, causing sustained downstream signalling by PI3K, and through MAPK-ERK promotes cell survival, migration, and invasion. Stabilization of FGF-1 receptor by N-cadherin, followed by MAPK-ERK activation, may result in increased transcription of the extracellular matrix-degrading enzyme MMP-9, and hence, an increased cellular invasiveness. The FGF-1 may interact with the fourth extracellular domain (EC4) of N-cadherin based on the fact that transfer of EC4 domain from N-cadherin onto E-cadherin reconstituted the invasive function of N-cadherin [21]. Other authors suggest the role of the cytoplasmic domain of N-cadherin [35, 36] and soluble N-cadherin domain in regulating cell migration [37].

Current results demonstrate the important role of N-cadherin in melanoma cell invasion. In light of our former studies, where observed was a significant reduction in melanoma cell proliferation in effect of N-cadherin silencing, the hypothesis that MMP-9 expression and cellular invasion are governed by at least two, but possibly more, distinct intracellular signalling pathways when stimulated by N-cadherin-FGFR signalling [21], seems to be a sound one.

Melanoma cells react differently to the MEK kinase inhibitors, as Ferguson et al. [22] observed a slight increase in the activity of MMP-2 in the use of ERK1/2 inhibitor—U126. Preclinical data presented by Catalanotti et al. [38] suggest that patients with B-RAF mutant melanomas and PI3K/AKT pathway activation are less sensitive to MEK inhibition. 50% of melanoma cases showed presence of B-RAF mutation. For example, all studied cell lines exhibit B-RAF mutations: Lu1205 and WM793—V600E, WM115 and WM266-4 V600D.

The need for search of effective multitargeted therapies or broad spectrum approach to cancer treatment [39] fully justifies the attempts to recognize the effect of collective inhibition of the activity of N-cadherin and some crucial signal transducers, such as protein kinases: PI3K, ERK, and mTOR. The conducted studies demonstrated that inhibition of N-cadherin or one of kinases produces less satisfactory effects than parallel inhibition of at least two of them, as shown here and formerly [23]. These results many open up new avenues for developing low-dose anticancer therapies, which would allow to reduce the negative side effects of the high doses often administered in case of individual anticancer agent [40].

Particularly promising are the results with regard to the decrease in melanoma cells invasiveness after N-cadherin gene silencing and the use of everolimus inhibitor of the mTOR pathway. everolimus is a low-toxicity drug that has been used as an immunosuppressant in organ transplant patients, in particular in cancer-related cases for several years [41]. In recent years, use of everolimus and other inhibitors of the mTOR pathway for antitumor therapy has attracted some interest [41, 42]. The fact that their inhibitory effect may be significantly enhanced by simultaneous use of siRNA for N-cadherin may be worth considering as a potentially new approach to effective treatment of melanoma.
